# Clinical, radiological, and pathological features of minute pulmonary meningothelial-like nodules and diffuse pulmonary meningotheliomatosis

**DOI:** 10.3389/fmed.2023.1209491

**Published:** 2023-07-26

**Authors:** Naijian Li, Sicui Quan, Qin Liu, Zhiling Xie, Qiong Wang, Nian Wang, Jinlin Wang

**Affiliations:** ^1^Pulmonary and Critical Care Medicine, State Key Laboratory of Respiratory Disease, National Center for Respiratory Medicine, Guangzhou Institute of Respiratory Health, The First Affiliated Hospital of Guangzhou Medical University, Guangzhou, China; ^2^Department of Pathology, National Center for Respiratory Medicine, Guangzhou Institute of Respiratory Health, The First Affiliated Hospital of Guangzhou Medical University, Guangzhou, China; ^3^Department of Pathology, He Xian Memorial Hospital of Panyu District, He Xian Memorial Affiliated Hospital of Southern Medical University, Guangzhou, China

**Keywords:** minute pulmonary meningothelial-like nodules, diffuse pulmonary meningotheliomatosis, clinical features, pathology, radiology

## Abstract

**Background:**

Minute pulmonary meningothelial-like nodules (MPMNs) and diffuse pulmonary meningotheliomatosis (DPM) are both rare lung diseases that involve the proliferation of cells of meningothelial origin in the lungs. However, few studies have focused on the clinical, pathological, and radiological features of MPMNs and DPMs.

**Methods:**

The clinicopathological data of 167 cases diagnosed as MPMNs and 13 cases diagnosed as DPM in the China National Center for Respiratory Medicine were examined. Based on clinical data, CT images, and morphological features, this study analyzed the similarities and differences between MPMNs and DPM.

**Results:**

The detection rates of MPMNs and DPM were 1.9 and 0.15%, respectively. Compared to MPMNs, DPM patients were all women (100% vs. 79.4%, *P* = 0.066), had a younger age (51.4 ± 7.7 vs. 57.9 ± 8.5, *P* < 0.01), and had higher pulmonary function (*P* < 0.01 or *P* < 0.05). The chest CT of DPM patients showed diffuse ground-glass opacity nodules measuring 2.0–8.0 mm in diameter, with the number of nodules ranging from 40 to >600 per lung. There were no significant differences in nodule volume [28.0 (12.1, 65.1) mm^3^ vs. 28.7 (17.1, 48.9) mm^3^, *P* = 0.451] and CT values [−646.8 (−732.5, −514.5) Hu vs. −588 (−674, −480) Hu, *P* = 0.215] between MPMNs and DPM. MPMNs are characterized by reactive hyperplasia pulmonary nodules, which can be solitary or multiple.

**Conclusion:**

This study suggests that there are many different characteristics between patients with MPMNs and DPM. The limited findings challenge the notion that DPM is a rare subtype of MPMNS.

## Introduction

Minute pulmonary meningothelial-like nodules (MPMNs) are a type of interstitial cell proliferation lesion that is inconspicuous and without obvious clinical symptoms (1, 2). They are usually discovered incidentally under a microscope or during imaging inspection for other reasons and can occur singly or multiply with diameters ranging from 1 to 8 mm. Immunohistochemistry, ultrastructure, and fluorescent *in situ* hybridization studies indicate that these nodules originate from the meningeal epithelium (3, 4). MPMNs are assumed to represent reactive changes in the lung tissue rather than a true neoplasm or tumor and do not require any specific treatment.

Diffuse pulmonary meningotheliomatosis (DPM) is a rare condition characterized by the diffuse proliferation of meningothelial cells within the lung parenchyma. DPM is a term coined by Suster in 2007, which is considered a rare type of MPMN with unclear clinical significance (5). It has been speculated to arise from the proliferation of MPMNs although the exact mechanism of transformation is not well understood. DPM typically presents with respiratory symptoms such as cough and shortness of breath, and it is more commonly discovered in women; many patients have a history of malignancy and are typically diagnosed by surgical lung biopsy (6, 7). As a rare disease, there have been few research reports on DPM, and clinical doctors, radiologists, and pathologists have only limited understanding of DPM.

This study aimed to investigate and compare the clinical, pathological, and radiological features of MPMNs and DPM and to further use these features to explain the formation mechanism of MPMNs and DPM. This will provide therapeutic insights for clinical practice.

## Materials and methods

### Study population and diagnostic criteria

This study used a retrospective case–control study design. From January 2018 to December 2022, a total of 8,847 patients received pulmonary resections for pulmonary nodules at the Department of Thoracic Surgery, National Center for Respiratory Medicine, Guangzhou, P.R., China. Pathological specimens of MPMNs were mainly recognized from two different scenarios. Most of the MPMNs were found incidentally in the background alveolar lung parenchyma near the lung nodules, and the other MPMNs were confirmed by pathology after surgical resection due to misdiagnosis.

After two senior pathologists blindly and independently rechecked the slides, according to the 2021 edition of the World Health Organization Classification of Lung Tumors (8), a total of 167 cases were diagnosed with MPMNs and 13 cases were diagnosed with DPM. Head CT or MR excluded intracranial/extracranial meningioma. All clinical information was collected according to standard procedures by the First Affiliated Hospital of Guangzhou Medical University. The Ethics Commission of the First Affiliated Hospital of Guangzhou Medical University approved the study. Informed consent was waived due to the retrospective nature of this study.

### CT examinations

The chest unenhanced CT scans were obtained mainly with a 256-row multidetector system (GE Revolution CT; GE Healthcare). According to commercially available computer-aided detection software (DeepWise, Hangzhou, China), radiological features were assessed independently and blindly by two chest radiologists. Radiological features included the location of nodules, nodule morphology, size, attenuation, volume, and quantity. National Medical Products Administration (NMPA) in China (Registration No. XZXK-2020-1570) has officially approved the clinical use of this system.

### Pathology analysis

The resected lobes were fixed in 4% neutral formaldehyde and embedded in paraffin, and 5-μm sections of the lobes were cut and stained with hematoxylin and eosin. The lesion histological features were observed with an optical microscope. Immunohistochemical (IHC) staining was performed using the following antibodies: cytokeratin (CK), CD56, epithelial membrane antigen (EMA), progesterone receptor (PR), thyroid transcription factor (TTF-1), and vimentin. All reagents and antibody staining were performed according to the manufacturer's instructions.

### Statistical analysis

To compare the frequencies among the MPMNs and DPM groups, the χ^2^ test and Mann–Whitney U-test were performed with the SPSS version 27 (IBM SPSS, Armonk, NY, USA). A *P*-value of <0.05 was considered to be statistically significant.

## Results

### Patient characteristics

In this study, 167 patients with MPMNs were identified among all 8,847 patients who received lung resections in our department of thoracic surgery, resulting in a detection rate of 1.9%. The 167 patients included 132 women (79.4%) and 35 men (20.6%) with a mean age of 57.9 and a mean BMI of 23.5. It was found that MPMNs mainly occurred in individuals aged 50–69 years (70.7%) and were more frequently present in the right lobes (70.7%). Only 13 patients (7.8%) had a history of smoking, while 154 patients (92.2%) were non-smokers. Primary lung adenocarcinoma was significantly associated with MPMNs, and a total of 139 patients (83.2%) had MPMNs combined with lung adenocarcinoma (see Table 1).

**Table 1:** Demographic and clinical characteristics of MPMNs and DPM patients.

**Clinical features**	**MPMNs**	**DPM**	***P-*value**
No. of patients	167	13	
Gender, women (%)	132 (79.4)	13 (100)	0.066
BMI, kg/m^2^	23.5 ± 3.2	22.6 ± 2.5	0.316
**Smoking status**			0.580
Current smoker, *n* (%)	10 (6.0)	0	
Ex-smoker, *n* (%)	3 (1.8)	0	
Never smoker, *n* (%)	154 (92.2)	13 (100)	
**Age (years)**	57.9 ± 8.5	51.4 ± 7.7	**<0.01**
30–49, *n* (%)	34 (20.4)	6 (46.2)	
50–69, *n* (%)	118 (70.7)	7 (53.8)	
Over 70, *n* (%)	15 (8.9)	0 (0)	
**Resected lobe**, ***n*** **(%)**			0.635
Right	118 (70.7)	8 (61.5)	
Left	49 (29.3)	5 (38.5)	
Lung adenocarcinoma, *n* (%)	139 (83.2)	10 (76.9)	0.842
**Pulmonary function test**			
FEV1 (L)	2.0 ± 0.5	2.6 ± 0.6	**<0.01**
FEV1%	87.1 ± 15.8	102.2 ± 9.2	**<0.01**
FVC (L)	2.8 ± 0.6	3.3 ± 0.8	**0.031**
FVC%	103.0 ± 18.0	111.8 ± 10.8	0.053
FEV1/FVC%	76.3 ± 6.6	77.5 ± 6.0	0.751

Data are shown as means ± SD. BMI, body mass index; FEV_1_, forced expiratory volume in 1 s; FVC, forced vital capacity. The bold values are indicated to highlight the *P* value <0.05 or 0.01.

There are many different characteristics between DPM patients and MPMNS patients. There were 13 cases of DPM in this study, and the detection rates of DPM were 0.15%, accounting for 7.8% of all MPMNs. All 13 patients with DPM were women (100% vs. 79.4%, *P* = 0.066) and had a younger age (51.4 ± 7.7 vs. 57.9 ± 8.5, *P* < 0.01). Furthermore, patients with DPM had higher forced expiratory volume in 1 s (2.0 ± 0.5 vs. 2.6 ± 0.6, *P* < 0.01) and forced vital capacity (2.8 ± 0.6 vs. 3.3 ± 0.8, *P* = 0.031) than patients with MPMNs. These results indicate that pulmonary function tests in DPM patients are normal. There was no significant difference in BMI, smoking status, or lung adenocarcinoma combined with the two groups. The clinical findings in 167 patients with MPMNs and 13 patients with DPM are summarized in Table 1.

### Radiological features

**Figure 1:**
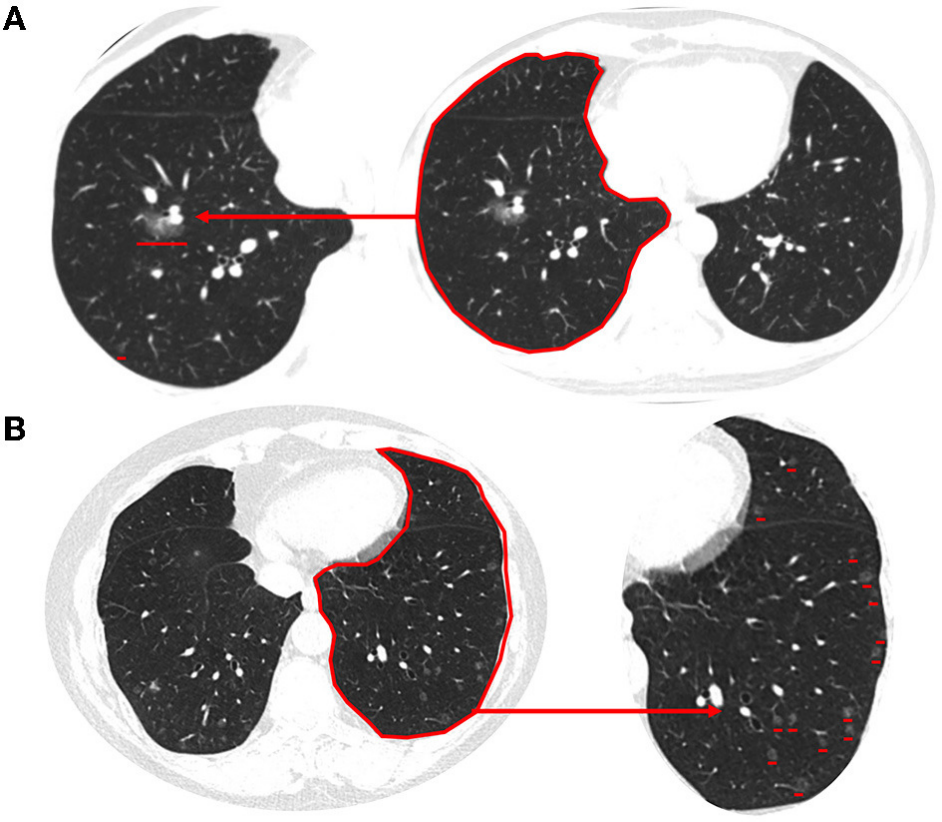
Typical imaging features of MPMNs and DPM on chest CT. **(A)** An MPMN located in the right lower lobe close to that of malignant pulmonary nodules presents as solid nodules on a chest CT scan. **(B)** Chest CT scan (lung window) showing diffuse multiple minute nodules distributed randomly throughout both lungs. These range in size from 2 to 5 mm, and some are more abundant in the subpleural region. The magnified image of the lower left lung shows a clearer image of multiple lung nodules.

MPMNs are asymptomatic solitary or multiple nodules that are usually noted incidentally on high-resolution chest CT scans performed for the detection of other malignant nodules. MPMNs may appear on chest CT as pure ground-glass opacity, mixed, or solid nodules, with sizes ranging from 1.0 to 8.0 mm. These nodules typically have a round shape and well-demarcated borders. The median and interquartile range of the number, volume, and CT value of pulmonary nodules in MPMNs patients were 3 (2, 5), 28.0 (12.1, 65.1) mm^3^, and −646.8 (−732.5, −514.5) Hu, respectively. The location of a typical MPMN nodule is close to that of malignant pulmonary nodules (Figure 1A).

High-resolution chest CT of DPM patients showed multiple bilateral reticulonodular infiltrates or ground-glass opacity nodules, measuring 2.0–8.0 mm in diameter, some of which were cavitary in nature. The nodules were present in both deep and subpleural locations, but they were typically more abundant in the subpleural region (Figure 1B). The data for the 13 patients with measurable nodules on CT scans are summarized in Table 2. According to computer-aided detection software and chest radiologists assessed, the number of nodules ranged from 40 to >600 per lung. The average volume and CT value of pulmonary nodules in DPM patients were 34.22 mm^3^ and −577 Hu, respectively. There were no significant differences in nodule volume and CT values between MPMNs and DPM. The distribution of nodules was more widespread in the right lung than in the left lung (54.3 ± 12.2 vs. 45.7 ± 12.2, *P* = 0.081). We conducted a 1-year postoperative follow-up on 13 patients, and the chest CTs showed no significant changes in the lesions.

**Table 2:** Radiological features of DPM.

**Patients**	**No. of nodules**	**Right lobe (%)**	**Left lobe (%)**	**Volume (mm^3^)**	**CT value (Hu)**
N1	640	275 (43.0)	365 (57.0)	29.8 (18.6, 47.8)	−612 (−689, −506)
N2	399	202 (50.6)	197 (49.4)	22.8 (13.1, 33.3)	−721 (−754, −685)
N3	184	96 (52.2)	88 (47.8)	49.3 (23.1,103.0)	−373 (−478, −178)
N4	135	66 (48.9)	69 (51.1)	26.5 (15.9, 48.9)	−735 (−766, −701)
N5	121	65 (53.7)	56 (46.3)	25.5 (17.0, 36.1)	−621 (−556, −668)
N6	76	45 (59.2)	31 (40.8)	24.8 (15.2, 36.3)	−574 (−633, −514)
N7	75	52 (69.3)	23 (30.7)	46.5 (35.4, 78.8)	−588 (−656, −405)
N8	67	40 (59.7)	27 (40.3)	17.1 (12.7, 24.1)	−540 (−595, −467)
N9	56	44 (78.6)	12 (21.4)	54.8 (28.7, 98.6)	−610 (−693, −480)
N10	55	26 (47.3)	29 (52.7)	21.3 (10.4, 67.0)	−580 (−627, −328)
N11	46	16 (34.8)	30 (65.2)	11.0 (8.8, 16.0)	−383 (−509, −164)
N12	42	28 (66.7)	14 (33.3)	86.4 (48.5,130.7)	−644 (−716, −584)
N13	41	17 (41.5)	24 (58.5)	29.0 (17.3, 50.0)	−523 (−674, −394)

CT value and volume are shown as median (interquartile range).

### Histological and immunohistochemical features

**Figure 2:**
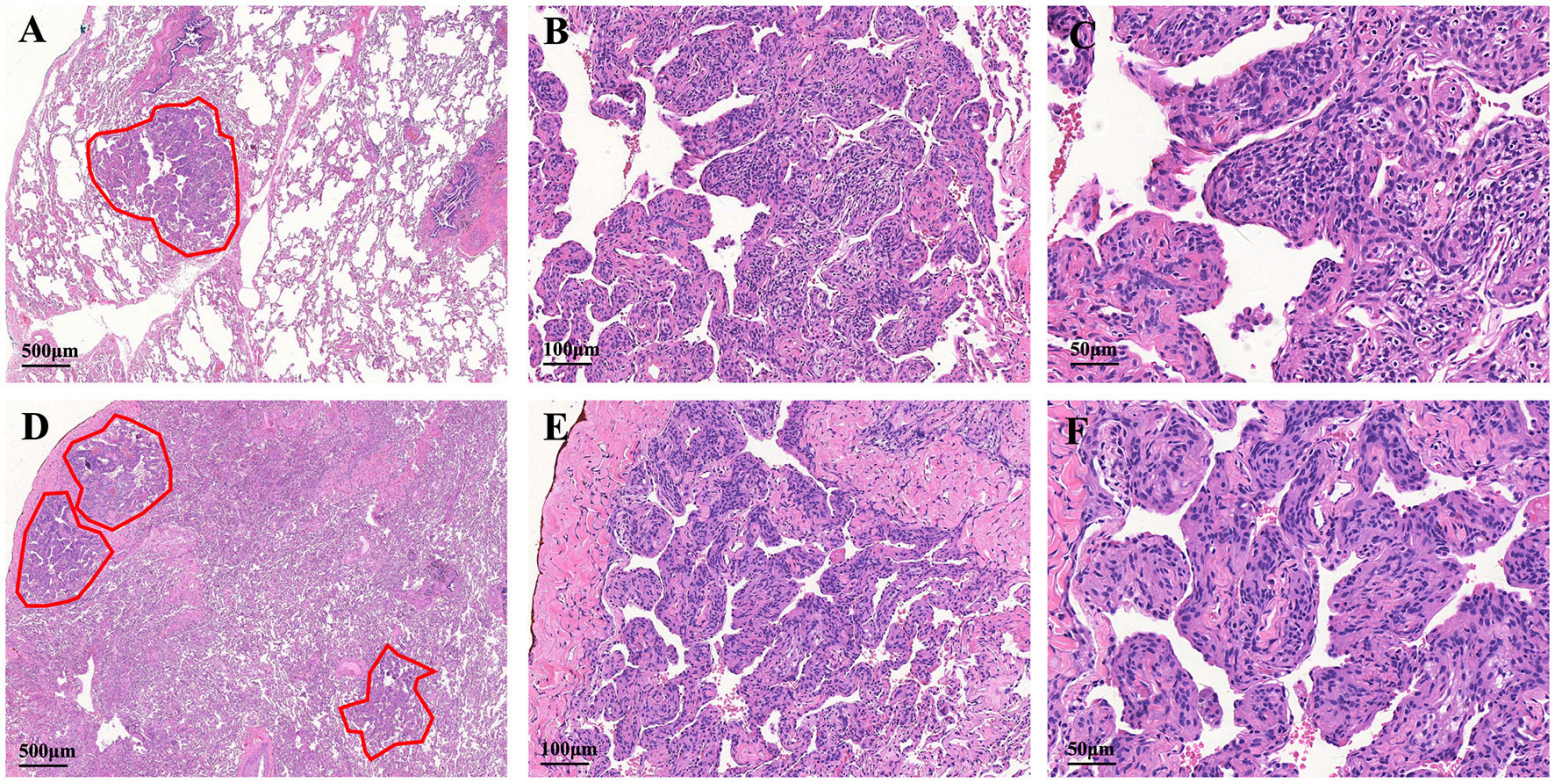
Histologic features of the MPMNs and DPM. **(A)** One meningothelial-like nodule in the left lower lung (outlines the lesion with a red line). **(B, C)** The cells are spindle- and oval shaped, with acidophilic cytoplasm and fine granular chromatin. The cell nucleus is elliptical with indistinct nucleoli, borders are not clear, and there is no dysplasia. **(D)** Low-power magnification shows multiple foci of meningothelial-like nodules. **(E)** Higher magnification shows well-circumscribed and nodular proliferation sharply demarcated from the surrounding pulmonary parenchyma. **(F)** Focal whorling of cells was observed within meningothelial-like nodules.

A diagnostic video-assisted thoracoscopic (VATS) lung wedge biopsy and histological analyses were performed for a definitive diagnosis. At low power field magnification, the hematoxylin and eosin stain of the lung tissue showed single small, clear, meningeal epithelioid nodules. Under the high magnification power field, the nodules arranged in nests were sharply demarcated from the surrounding pulmonary parenchyma. The cell nuclei are elliptical in shape, without obvious nuclear atypia. Some nuclei have very small nucleoli and no apparent mitotic figures (Figures 2A–C).

**Figure 3:**
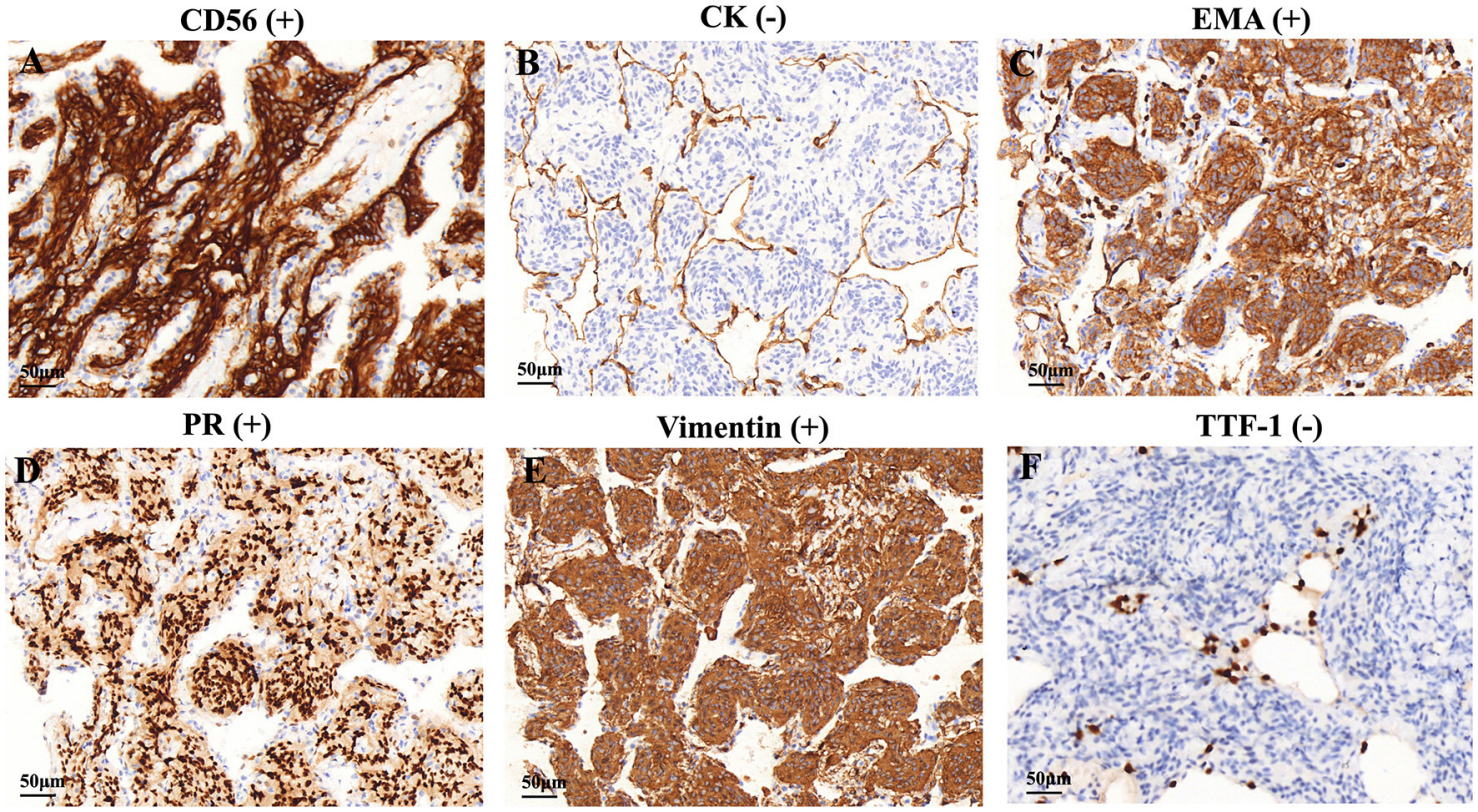
Immunohistochemical features of the DPM. Immunohistochemical staining showed that cells were positive for **(A)** CD56, **(C)** EMA, **(D)** PR, and **(E)** vimentin, negative for **(B)** CK and **(F)** TTF-1. CK, cytokeratin; EMA, epithelial membrane antigen, PR, progesterone receptor, TTF-1, thyroid transcription factor.

The specimens from DPM usually contained 3 or 5 meningothelial nodules per slide. However, some cases showed up to 10 discrete nodules per histologic section. In addition to exhibiting the pathological features of MPMNs, focal whorling of cells was observed in some areas when viewed at high magnification (Figures 2D–F). In total, 13 specimens were tested immunohistochemically. All 13 specimens (100%) tested positive for EMA, PR, and vimentin, 10 out of 13 specimens (76.9%) tested positive for CD56, and 7 out of 13 specimens (53.9%) tested positive for TTF-1, while 3 out of 13 specimens (23.1%) tested positive for CK (Table 3 and Figure 3).

**Table 3:** Immunohistochemical results of DPM.

**Patients**	**CK**	**CD56**	**EMA**	**PR**	**TTF-1**	**Vimentin**
N1	–	–	+	+	+	+
N2	–	–	+	+	+	+
N3	–	+	+	+	+	+
N4	–	+	+	+	+	+
N5	–	–	+	+	+	+
N6	+	+	+	+	+	+
N7	–	+	+	+	–	+
N8	–	+	+	+	–	+
N9	–	+	+	+	–	+
N10	–	+	+	+	–	+
N11	–	+	+	+	–	+
N12	+	+	+	+	–	+
N13	+	+	+	+	+	+

CK, cytokeratin; EMA, epithelial membrane antigen; PR, progesterone receptor; TTF-1, thyroid transcription factor-1; –, negative; +, positive.

## Discussion

In 1960, MPMNs were first reported by Korn et al. and were initially interpreted as chemoreceptors on the basis of their morphologic features and their close relationship with blood vessels (1). However, subsequent ultrastructural and immunohistochemical analysis revealed a strong similarity to meningothelial cells, leading to their reclassification as MPMNs (9, 10). Previous research has demonstrated that the autopsy-based detection rate of MPMNs ranges from 0.07 to 4.9%, whereas the detection rate of surgically excised specimens ranges from 0.6 to 7.0% (4, 11). This suggests that the incidence of MPMNs may be underreported, and the actual occurrence of this disease may be higher than previously estimated.

Our study reported clinical, pathological, and radiological features of 167 cases of MPMN. Individuals with MPMNs typically do not display any symptoms or may experience non-specific respiratory symptoms, as described in the aforementioned case. These nodules are usually identified as isolated pulmonary nodules on chest CT scans performed for other purposes or may be discovered incidentally during lung surgery. The primary reason for surgery in most patients with MPMNs was the detection of other malignant nodules on chest CT scans rather than the MPMNs (12, 13). Therefore, the removal of the MPMNs was incidental, which explains why the majority of patients' primary diagnosis was lung adenocarcinoma.

DPM is a rare type of MPMN and was first reported by Suster et al. in 2007 (5). We conducted a review of the literature through March 2023 for studies reporting cases of DPM by searching multiple scholarly databases (6, 7, 14–17). Of the 48 cases identified, only 5 were men (10.4 %), and 43 were women (89.6%). The age of onset varies from 30 to 80 years old, with an average age of 59.7 years at diagnosis. Thirty-eight cases (79.2%) had no obvious symptoms, and radiological abnormalities were found incidentally. Only 10 cases (20.8%) sought medical attention due to respiratory discomfort, and only 8 patients (16.7%) were confirmed by bronchoscopic biopsy, while the remaining patients were diagnosed by surgical lung biopsy.

Although DPM is considered a rare subtype of MPMNs, limited research has challenged this view. First, the genetic analysis found that only a small number of cells in solitary MPMNs had single-gene locus mutations, and no multi-gene locus losses were found, suggesting that they are not tumorous and are more similar to benign reactive proliferation. In contrast, DPM has an increased proportion of single-gene locus mutations and exhibits multi-gene locus loss heterogeneity (9, 18). This indicates that DPM has significantly increased instability compared to MPMNs, representing a transitional state from reactive proliferation to tumorigenesis. Second, chest CT of DPM patients showed diffuse and micronodular/miliariform patterns, which are inconsistent with the characteristic of MPMNs being isolated and reactive hyperplasia pulmonary nodules. Third, in this study, we found that all patients diagnosed with DPM were women, had a younger age of onset, and had normal ranges of FEV1 and FVC.

We have summarized eight diagnostic points of DPM, and we have presented further explanations on these features: (1). Gender and age characteristics of DPM patients: studies have shown that DPM is more commonly diagnosed in women than men (up to 12:1), and the onset age of most patients is between 45 and 60 years old. (2). Smoking and occupational dust exposure history: Most DPM patients do not have a history of smoking or occupational dust exposure. (3). Symptoms: Most DPM patients do not have significant symptoms, but some patients may experience unexplained respiratory symptoms such as chest tightness or cough. (4). Lung function, tumor markers, and immune indicators: DPM patients usually perform normally in these indicators, which can help doctors rule out other lung diseases. (5). Chest CT: Chest CT of DPM patients typically shows diffuse multiple ground-glass or cystic nodules in both lungs, with a number ranging from 40 to over 600 per lung, with a diameter of 1–8 mm. Lung-RADS classification is mainly type 2. (6). Complications: DPM patients are prone to concurrent lung adenocarcinoma and therefore require a regular follow-up. (7). Diagnostic methods: VATS is one of the main diagnostic methods for DPM currently. (8). Pathological features: The pathological features of DPM mainly manifest as multiple visible MPMNs under the microscope.

We acknowledge the following limitations of the present study. First, this study is a single-center retrospective study with a small sample size, and selection bias may exist. Second, the data were collected retrospectively, and long-term results cannot be obtained. Third, minute pulmonary meningothelial-like nodules and diffuse pulmonary meningotheliomatosis are rare diseases that require a high level of expertise from pathologists, and there may be a certain degree of misdiagnosis.

## Conclusion

In summary, our study reported the clinical, pathological, and radiological features of 167 cases of MPMNs and 13 cases of DPM. MPMNs present as single or multiple ground-glass nodules that are found more often in female patients with lung adenocarcinoma. DPM is a rare disease more commonly encountered in women and is characterized by randomly diffuse bilateral interstitial nodular lung infiltrates. The differences and pathogenesis of MPMNs and DPM need further investigation in future.

## Data availability statement

The raw data supporting the conclusions of this article will be made available by the authors, without undue reservation.

## Ethics statement

The studies involving human participants were reviewed and approved by Ethics Committee of the First Affiliated Hospital of Guangzhou Medical University. Written informed consent for participation was not required for this study in accordance with the national legislation and the institutional requirements.

## Author contributions

NL, JW, and SQ conceived the study, directed the project, and designed the experiments. ZX, QW, NW, and QL obtained the samples and clinical information, interpreted the results, and wrote the manuscript. All authors read and approved the final manuscript.
